# Employees, Advisees, and Emerging Scholars: A Qualitative Analysis of Graduate Students' Roles and Experiences of Sexual Violence and Sexual Harassment on College Campuses

**DOI:** 10.1007/s12119-021-09841-w

**Published:** 2021-03-20

**Authors:** Brittnie E. Bloom, Cierra Raine Sorin, Jennifer A. Wagman, Laury Oaks

**Affiliations:** 1 University of California San Diego Joint Doctoral program in Public Health, San Diego State University Graduate Program in Public Health, San Diego, CA USA; 2 University of California Global Health Institute Women’s Health, Gender, and Empowerment Center of Expertise, Los Angeles, CA USA; 3 Department of Sociology, University of California Santa Barbara, Santa Barbara, CA USA; 4 Department of Community Health Sciences, Fielding School of Public Health, University of California Los Angeles, Los Angeles, CA USA; 5 Department of Feminist Studies, University of California Santa Barbara, Santa Barbara, CA USA

**Keywords:** Sexual assault, Sexual harassment, Graduate students, Campus climate, Power differences, Diversity

## Abstract

Sexual violence and sexual harassment (SVSH) are pervasive across university campuses. SVSH research rarely focuses on graduate students, who occupy unique positions within university settings due to their multiple responsibilities (e.g., teaching, research, mentoring), including managing unequal power dynamics with mentors and advisors. As part of a larger qualitative study, we sought to better understand SVSH generally and, when applicable, experiences of SVSH among graduate students on three campuses. Our primary research questions were: (a) How graduate students understand SVSH on their campus and whether they are at risk, (b) How graduate students navigate power dynamics that position them to experience SVSH, and (c) What graduate student-centered solutions and improvements can make campuses safer, more equitable spaces for all students. To answer these questions, we conducted 21 in-depth interviews and 8 focus group discussions with a diverse group of graduate students across various graduate programs. Using grounded theory and implementing student-centered approaches to data collection and analysis, we identified multiple themes, including graduate students’ uncertainty regarding SVSH on campus; and how power relations with faculty, combined with distrust of university processes, keep many graduate students silent about SVSH. Finally, employing graduate students’ suggestions, we offer recommendations for how universities can improve campus climate and SVSH resources for graduate students.

## Introduction

### A Critique of Current University-Based SVSH Research

Studies on college sexual violence and sexual harassment (SVSH) have grown substantially in the last decade; nevertheless, SVSH remains a significant problem in college settings. SVSH, a term commonly used in campus-based violence work, encompasses a number of behaviors that include, but are not limited to, penetrative sexual assault, non-penetrative sexual assault, relationship violence, stalking, sexual harassment and invasions of sexual privacy (UC Office of the President Sexual Violence and Sexual Harassment Policy, 2020). The growing concern and attention focused on SVSH on college campuses, coupled with the #MeToo movement (Kessler et al., 2020; Kumar & Verma, 2020; Murphy, 2019), has increased pressure on universities to address and decrease SVSH (Edelman, 2018). However, the majority of research, student outreach and service provision remains focused on undergraduate students, leaving graduate students’ experiences of SVSH unheard and undocumented (Hirsch & Khan, 2020; McMahon et al., 2018). Racial, economic and gender disparities are increasingly visible at the graduate level (Creighton, 2007; Hoffman et al., 2018; Hyun et al., 2006; Johnson-Bailey, 2004; Wohlgemuth et al., 2007), and in addition to being at higher risk of experiencing SVSH (Bonistall Postel, 2017; Bonomi et al., 2018; Coulter & Rankin, 2020; Coulter et al., 2017; de Heer & Jones, 2017; Griner, 2017; Martin et al., 2011), graduate students who face overlapping disparities due to institutional racism, classism, sexism, ableism and heterosexism may encounter significant barriers to identifying support and resources related to experiences of SVSH. Recent studies that have included graduate students found that 5.2% experienced sexual assault since attending their university (McMahon et al., 2018), 38% of women and 23% of men experienced sexual harassment from a faculty or staff member (Rosenthal et al., 2016), 57% of women and 38% of men experienced sexual harassment from another student (Rosenthal et al., 2016) and 13% had a peer disclose that they had experienced SVSH (McMahon et al., 2018). In 2015, the University of Virginia released a report on SVSH, concluding that compared to their male counterparts, female graduate students were six times more likely to experience non-consensual penetration or sexual touching (Cantor et al., 2015).

The structure of academia generally and graduate education specifically creates and maintains close relationships complicated by power hierarchies and the abuse of power, which has been highlighted at length in grey literature, citing mental health crises, infringement of boundaries by mentors and program heads (including sexual violence and harassment), and defunct labor and SVSH reporting policies (Gessen, 2018; Perry, 2018; Wedemeyer-Strombel, 2018; Woo, 2019; Yesko, 2014), but is noticeably lacking from academic journals and peer-reviewed publications. Further, graduate students have unique risk factors that require more focused attention to prevent SVSH. Such factors include having less knowledge of campus resources and confidence in where to seek assistance, problematic power dynamics with their mentors, and social isolation (Cantalupo & Kidder, 2017; McMahon et al., 2018; Noy & Ray, 2012). Less cited factors include graduate students’ investment in their professional identities, the “small world” of academic disciplines and departments, as well as specific funding and other professional and personal opportunities that are tied to departments, mentors and/or advisors (Rosenthal et al., 2016). Being a graduate student necessitates participating in non-formal spaces, including student-mentor office hours, conferences, email and other online interactions, social events, and happy hours. These spaces often have unclear boundaries and can place students in situations where experiencing SVSH is more likely (Jackson, 2019). Risk for SVSH may be amplified in certain academic disciplines more than others, including disciplines centered in STEM, which historically lack representation from non-cisgender, non-white, non-heterosexual and non-disabled faculty members (National Science Foundation & National Center for Science and Engineering Statistics, 2019; Shen, 2013). Further work highlighting these perspectives – from graduate students themselves – is necessary to adequately address graduate student experiences of SVSH.

### Elevating Graduate Students’ Voices to Fill Gaps and Identify Solutions

Elevating students’ voices has been used in high school and secondary education settings to improve campus climate and create partnerships between decision-makers (e.g., administrators) and students (Mitra, 2003). The majority of educational policies and decision-making is made on behalf of students instead of alongside them, leaving them without an invitation to the “proverbial decision-making table” (Lac & Cumings Mansfield, 2018). Highlighting and incorporating student voices in higher-education settings is growing in popularity (Cook-Sather, 2007; Mitra, 2008; Mitra et al., 2012), but has not been well-implemented within university settings—especially among graduate student populations. Though graduate students may be invited to participate in campus discussions of SVSH prevention, response, and support, specific considerations of their unique needs are often absent or ignored, as evidenced by the lack of graduate-specific programming for SVSH on university and college campuses.

Graduate students’ experiences and perspectives should be included in efforts to improve SVSH programs and policies. This qualitative study fills a unique gap in the literature on SVSH in university settings by elevating the voices of a diverse sample of graduate students. For the purposes of this analysis, we aimed to answer the following research questions: How do graduate students understand SVSH on their campus? How do graduate students navigate and negotiate power dynamics that uniquely position them to experience SVSH? And what graduate student-centered solutions and improvements can be made to make campuses safer, more equitable spaces for all students?

## Methods

This data was collected as part of UC Speaks Up, a multi-campus qualitative study led by faculty, staff, and students across three University of California (UC) campuses: UC Los Angeles (UCLA), UC San Diego (UCSD), UC Santa Barbara (UCSB) (UC Speaks Up, 2019). All research staff received comprehensive training in research ethics and compliance, qualitative research methods and trauma-informed care. Between January and May 2019, 21 in-depth interviews (IDIs) and 8 focus group discussions (FGDs) consisting solely of graduate students were conducted across the campuses by six graduate student research interns. Recruitment involved a combination of targeted and snowball sampling (a type of non-random, purposive recruitment methodology commonly used in qualitative research to reach and recruit difficult to reach and “hidden populations”) (Mack et al., 2005; Naderifar et al., 2017), including focused and mass email campaigns and posting and distributing flyers across campuses. All interested individuals completed a short screening survey online to assess eligibility for inclusion (English-speaking graduate students currently enrolled in a graduate-level program at UCLA, UCSD or UCSB, aged 18 or older). A subset of eligible participants was selected based on key demographics (gender, sexual orientation, race / ethnicity, type of graduate program) to attempt achieving representation at the student- and campus-level. Selected students were connected with a trained graduate student researcher to schedule a date, time and location for their participation in an IDI or FGD. Data collection occurred on campus in accessible and convenient locations where privacy could be ensured. All students provided written, informed consent to participate in the study and have the IDI or FGD audio recorded and received a $25 Visa gift card as compensation for their time. The study protocol was approved by the UCSD Human Research Protection Program, with reliance approval from the institutional review boards (IRB) at UCLA and UCSB.

Before engaging in the data collection process, student participants were informed about the topic of the study: sexual violence—the broadest umbrella term used in the field that also encompasses many increasingly specific terms (Rutherford et al., 2007). As is common in qualitative studies and in the design of IDI and FDG guides, conceptual definitions of specific phenomena may contribute to the end product, and reflects the intent to have the meaning of concepts defined by those being studied (DeCarlo, 2018). Twenty-one graduate students enrolled in various graduate and professional programs participated in an individual IDI. We invited participants to provide their own definitions of “sexual assault”, “sexual harassment” and “dating violence” before asking questions about sexual violence and/or sexual harassment. An analysis of these definitions is beyond the scope of this paper, and is being analyzed by other members of the larger research project. In addition to understanding students’ definitions of sexual assault, sexual harassment and dating violence, IDIs also aimed to explore students’ awareness of available services, prevention programs, and/or policies addressing SVSH at the university. Mobilizing a student-centered approach to this project, we were interested in how participants thought students could become more involved in preventing and responding to sexual and gender-based violence, and how the university could improve its services.

In addition to IDIs, a total of 43 graduate students participated across eight FGDs; the smallest group was three and the largest was seven. Each discussion was facilitated by a graduate student moderator, with an undergraduate or graduate student note-taker. The primary FGD topics included perceptions of sexual violence as a campus problem; the role that graduate students play in reporting and/or responding to sexual violence; and knowledge and opinions of on-campus prevention and response resources.

IDIs and FGDs were transcribed verbatim from audio recordings and written notes. Utilizing Dedoose (version 8.3.17), the authors followed an inductive grounded theory approach (Charmaz, 2006) in coding and analyzing the data to make sense of graduates students’ experiences and knowledge of SVSH and campus resources. Using a shared theme-matrix, the authors completed multiple iteratives of coding, resulting in the themes presented in the results section.

## Results

Sixteen Masters students, 23 Ph.D. students and 4 Professional students (i.e., law, dentistry, medical) participated. Students ranged in age from 23 to 34 years. Most participants identified as cisgender women or men, and four identified as transgender, non-binary or genderqueer. Participants were largely heterosexual and 32% identified as LGBTQ + . Similar to the demographics of UC graduate students (University of California, 2020), 41% identified as white, 25% as Asian, 16% as Hispanic/Latino, 6% as Black and 10% as more than one race or ethnicity. In order to protect the identities of the participants, who sometimes revealed personal experiences of SVSH, minimal demographic information is displayed; when it is provided, it is intended to provide context to a quote or conversation.

### How Graduate Students Understand SVSH on Their Campus and Whether They are At-Risk

When asked, “Is sexual violence and harassment a problem on your campus?”, graduate students provided mixed responses: while some students shared they were ill-equipped to respond about the prevalence or severity of SVSH on their campuses due to reasons including lacking and insufficient publicly-available data, others stood firm in their belief that SVSH is a problem on their campus and that graduate students are at significant risk.

“My answer is ‘I don't know’ because there is not sufficient information dissemination for me to know… As a graduate student I’m in a very isolated community. Obviously, I can’t say it’s not an issue because of my research institute [where] there are decades of history of a professor that was guilty of [SVSH]. So - I can’t say it’s not an issue.”

Students repeatedly focused on graduate students’ experiences of isolation, including feeling socially, emotionally and physically removed from the general student body and campus. A significant number of graduate students juxtaposed their experiences with those of undergraduates or their own memories of being an undergraduate. One participant indicated graduate students are “more spread out” and have less interaction with their colleagues. This may give graduate students a false sense of security or safety on campus, ultimately allowing them to falsely perceive themselves at decreased risk of experiencing SVSH.

### Graduate Students’ Unique Vulnerabilities and Inducements to Silence about SVSH

#### Graduate Students’ Multiple Roles and Power Dynamics with Faculty

The relationships graduate students have with mentors and professors are significant proponents of their vulnerabilities to experiencing sexual violence. In graduate school, students are simultaneously independent, budding experts in their field and are also dependent on mentors for learning, funding, publication and presentation opportunities, and personal and professional development. As shared by graduate students, this can culminate in confusing and uncomfortable roles and responsibilities, such that graduate students are often caught in grey areas with problematic power dynamics between themselves and their mentors. Students are uncertain about what the overall graduate school environment *should* look like, and are equally as unclear about their relationships with peers, faculty members and mentors alike, as they often are significantly different from their undergraduate experience, as one participant highlighted:

“I think especially in graduate school it’s really complicated because - you’re a student, but you’re not a student. You’re staff, but you’re not really staff. You’re a colleague with a professor, but not really. You work with them as an equal, but not really. Everything is so grey in grad school. I feel like it makes it even more complicated and breeds a certain environment that invites a lot of inappropriate behavior because it’s casual, but professional. Like, what is it? Am I going out for beers with the professor, or am I lower than you?”

Across campuses, students shared the difficulties they experience while navigating unclear roles and responsibilities. One student said that this brought up feelings of vulnerability, especially regarding reporting SVSH:

“I think graduate students are really vulnerable at [campus redacted] because we’re in a position of being laborers that aren’t really recognized as employees. We only have access to a certain amount of training, and our colleagues and peers are frequently our superiors. The people that we’re getting intellectual advice from are also our bosses in the classroom. They sit in a room together and determine our funding. There's so much more risk about reporting [SVSH].”

#### Culture Clash and Power Dynamics Leading to SVSH

Though graduate students reported experiencing SVSH from their peers, the majority experienced SVSH from a faculty mentor or advisor; many shared their personal stories and/or those of their peers. A handful of students from underrepresented backgrounds shared how their cultural practices contribute to their vulnerability of experiencing SVSH, which challenged their ability to gauge where appropriate boundaries should be drawn in what can be an already confusing environment. Specifically, in one FGD, several female Latinx students shared such experiences, including retellings of a male faculty member attempting to kiss them at a professional off-campus retreat, and a male professor who would make inappropriate comments about the graduate student’s body. Another Latinx-identifying student shared discomfort and confusion about having *their own* cultural practices of greeting and professionalism at odds with each other:

“I feel like I have to be professional, but then - should I refuse?... I am so uncomfortable when I go to a professor’s office who is Latino and they’re like, ‘Oh hey’ and give me a kiss on the cheek… Unfortunately, there’s a lot of professors who are Latino that do that with their female students... I think in Title IX trainings that we would talk about these cultural differences; that it’s never okay unless you have some kind of consent from the student. I think it’s completely inappropriate for a professor to be kissing the student, even if it’s on the cheek.”

This demonstrates that a widespread cultural practice (i.e., greeting with a kiss), may be received as a breach of professional boundaries and obfuscate consent, even by Latinx students themselves who understand the practice. This is a controversial subject publicly debated in various cultural contexts (Cavico et al. 2015; Richardson & Taylor, 2009; Sigal et al., 2005; Zimbroff, 2007) and, as the participant noted, calls for clarity of Title IX training and individual behavior change.

Another student called graduate school a “marginalized space without a lot of oversight” that lacks “modeling of healthy behavior” leading to “a lot of potential for abuse of power.” This student reflected on how power differentials that exist between graduate students and those they work with can create barriers to help-seeking. In their personal narrative, this participant experienced institutional betrayal (Smith & Freyd, 2013) related to their SVSH experience, ultimately believing their university (and the multi-campus university system) “doesn’t deal with sexual predators in a way that protects survivors or prevents future violence.”

#### Accountability and Institutional Distrust within the University System

Graduate students shared a unifying sentiment that accountability for perpetrators, most often referring to faculty, was lacking or nonexistent. Multiple graduate students reflected on their personal experiences of SVSH or their colleagues’ and how the university responded. One student focused on Title IX’s inability to punish or remove faculty for inappropriate behaviors:

“Currently Title IX can’t really do that much to faculty… Institutionally it’s so incredibly hard to hold the faculty accountable as individuals, laborers, teachers… especially once they have tenure.”

This student continued, speaking about a faculty member who had a Title IX report filed against them, was accused and found guilty of SVSH, but was never fired. In addition to wishing there was a public mechanism for students to express why they chose “not to work with this person and why [the university] is losing them [the student] as a scholar and a degree candidate,” and to protect future students from matriculating into the university with the same mentor, the student expressed how difficult it is to make progress in their graduate program if they are at odds with their mentor (i.e., the decision-maker and holder of their degree) and/or their university:

“Once we’re already in [graduate school], it’s really hard. I mean, I need to get out. I need to finish. I want my Ph.D. and I need the signature. You need institutional support to do that.”

Lacking institutional support, trust and confidence in the university system, students felt fearful and uncertain in their ability to succeed as graduate students and complete their degrees, especially international graduate students and those who identify as female. This was captured by a graduate student who shared their experiences of unequal power dynamics, reflecting on the actions of one faculty member who was known to target female international students:

“They had a very old-school bunch of faculty - old white dudes that took advantage of their positions of power in terms of how funding works, how research gets conducted, and even influencing what papers could get published, which greatly affected a lot of the female colleagues. There was this one faculty that was known to hire international postdocs from Europe. They became isolated in the US and when they were assaulted or harassed by their boss, they didn’t know what to do and were terrified because they were hoping to build a career in America and not get sent back because they’re here on visas.”

The student concluded by reporting that this faculty member was reported to human resources (HR), but ultimately never faced charges.

Encouraging faculty to be more engaged with graduate students and SVSH was another suggestion the graduate students provided. This is in alignment with other students’ retellings of positive experiences they have had with faculty. One Ph.D. student explained, “A more effective way [to increase accountability is] something with faculty. If you’re a grad student here for six years, maybe you make sure that someone in their department can spend one-on-one time with that person” to support having multi-level support systems in place and increased transparency between mentors and their graduate students. This student shared that having a single person largely responsible for the success of a graduate student is not an appropriate role for a faculty member to have without a system of checks and balances put in place. Moreover, recentering the role of mentorship - not only at the individual faculty level or within departments -  as a priority for the entire university community would help to ensure that these individual relationships, which can invite abuses of power, are only one way graduate students find support in the university. Ensuring that there is a web of support available can help dismantle the problematic power structures as they currently exist and protect graduate students from SVSH.

These suggestions coincide with two questions raised by a graduate student in an all-male focus group: “I think it speaks to this larger conversation about when you do something that’s harmful to someone else, who are you held accountable to?” Emphasizing the need for policies that “really focus on community accountability,” he continued:

“You can say what you will about changing an institution and how that’s actually effectively done, but it starts with people who are invested in your university and with graduate students, undergrads, and faculty. If there’s a faculty member who was a problem, then don’t get that faculty member on board… You [can talk] all you want, but your actions at the end of the day will speak louder than your words. And if you don’t effectively call out people for doing things that are further propagating these problems, then in my mind you have no accountability and therefore, I don’t see you as a solution, I see [you] as a problem.”

#### Uncertainty in Reporting Processes

Graduate students’ inability to trust university leadership to appropriately listen and respond to their experiences of SVSH further perpetuates disparities that exist in officially reporting help-seeking for experiences of SVSH. Students provided many accounts of how the tight-knit academic community, the grey areas of professional and personal relationships in graduate school, and the multiple roles that graduate students play interact with each other to create high levels of distrust in reporting and help-seeking processes:

“There’s no institutional frame around our relationship as laborers and advisees and intellectuals. We can’t be shepherded into the field without intellectual superiors drawing us in. There’s a huge risk of reporting, and… I don't trust Title IX to handle my grievance in a way that’s going to keep me private in a really small community. We all know - we all hear the stories.”

The deep-seated skepticism about whether university and legal systems adequately protect graduate students and their anonymity, unfortunately, creates additional burdens for graduate students—specifically in their non-academic roles as a peer, friend, colleague and/or advocate. One student discussed how a handful of their peers had come to them for advice and resources related to their experiences of SVSH. In addition to the burden graduate students experience as laborers, teachers and researchers, this particular graduate student was also put in the position to serve as an advocate and confidant.

“I had graduate students come to me with instances of sexual harassment and sexism, and I tried to get more information on their behalf and connect with resources… They felt really uncomfortable because they were terrified about it coming back to the faculty they were working for... It was so hard to get help while being anonymous.”

### Shifting SVSH Culture to Make Campuses Safer for Graduate Students

#### SVSH as Diversity Work on Campuses

SVSH affects all members of a university campus, but most obviously impacts women, people of color, and queer individuals, in part due to policy and problematic social norms which create increased vulnerability associated with marginalized identities. Thus, combatting SVSH is not just an issue of justice, but one of equity that is necessary in academia’s purported commitment to diversity and inclusion, as many graduate students alluded to. This further emphasizes the significant amount of additional labor that fighting for change on campuses includes; labor that is often taken on by overworked graduate students, who are often themselves survivors of SVSH:

“Being a higher ed researcher, I still have some naive hope that student organization can lead to change. I recognize that when I say ‘organizing’, I imply that I am expecting women and femmes and queers of color that are survivors to lead this emotional labor front, and that is not fair on them... But in an ideal world, every single students’ voice at this campus holds equal weight, and finding ways to force administrators to remember that is a way to lead to change.”

#### Cultural and Institutional Shifts are Needed

After sharing their personal and shared experiences of SVSH as graduate students, participants provided a multitude of ideas focused on changing campus culture. One student shared that they “haven’t talked to anyone on this campus [who has identified themselves to me as a survivor] who feels like they’re supported by the university,” which suggested a “deep-rooted cultural problem” necessitating systemic change. Many graduate students are deeply aware of how their identity as a marginalized or multiply-marginalized student makes them increasingly vulnerable to experiences of SVSH, and also that the education, prevention and service options provided by the university are not tailored or specific to them or their unique needs. In alignment with this, one specific solution that graduate students advocated for was altering the prevention and education training for SVSH (specifically for faculty and staff) across departments to both go beyond simple online modules and to redirect the content to be centered “around identity, privilege and oppression.” SVSH is inherently linked to multiple sites of power and oppression, and the lack of an intersectional approach to prevention and response on these campuses was cited as detrimental:

“As a Latina, I don’t feel that the training on sexual violence, or in general the approach that the university takes, is really cater[ing] to the experiences of diverse groups. I feel like it’s a one-size fits-all and we experience violence very differently, even within the #MeToo movement. It hasn’t really been as inclusive to [all] people, as people of color experience [sexual violence] differently… The university-wide response has been inadequate, especially for people of color.”

Creating greater community buy-in is also important to changing campus culture and increasing reporting and help-seeking. Many graduate students focused on the need to create “survivor-centered processes” within their university where survivors will be supported:

“The Campus Advocacy Resource and Education (CARE) Centers might help survivors get therapy resources, but it doesn’t deal with the fact that if you are victimized by somebody on campus - they’re still here. There is no... restorative [justice] process. It really feels like survivors are not supported here or believed, and people aren’t gonna report under those conditions.”

#### Reframing Survivorship and Supporting Survivors

It is important for universities to consider preventing and responding to SVSH as diversity work, beyond Title IX’s existing and often questionable role in protecting the university over students. The oft-invisible nature of survivorship means that students who stop making progress towards their degrees, sometimes leaving their programs or academia altogether, may not be recognized as students in need of aid. One graduate student emphasized how the university should reconsider its focus on SVSH for the sake of supporting survivors of SVSH:

“For the record, people who have survived or are [experiencing SVSH] on campus are probably also some of the best researchers and the best teachers, but because they’re dealing with this, they’re falling behind and falling through the cracks… and it’s really hidden… We see students of color and women falling through the cracks. But it's harder to see this because [being a survivor is] a hidden status. The university is fucking up.”

One student shared that the most appropriate first step in caring for survivors is the university leadership “simply talking to survivors,” but that something as simple as that is “so beyond what I see the UC ever doing.” Ultimately, students want to see a shift in how survivors are viewed in academia and to have a voice in sharing what survivors want from university leadership.

## Discussion

Despite growing efforts to combat SVSH on university campuses in recent years (Edelman, 2018; Murphy, 2019), the majority of research has focused on undergraduate students (Hirsch & Khan, 2020; McMahon et al., 2018). Graduate students have unique experiences of SVSH, which differ substantially from those of undergraduate students (McMahon et al., 2018). These differences have long gone under-documented, despite the fact that graduate students are often on campus longer than undergraduates and are essential to the success of many universities. Supporting our findings, prior research has revealed that graduate students, especially those who belong to marginalized or multiply-marginalized student groups, and/or occupy multiple positions within the university system, are uniquely vulnerable to SVSH. Targeted policy, prevention and services has the potential to have significant and positive outcomes related to preventing SVSH (Gialopsos, 2017); nevertheless, graduate students are not often considered or invited to the “decision-making table” regarding campus-based efforts to address SVSH. Further, the graduate students in our study reported experiences of institutional betrayal (Otsri, 2020; Smith & Freyd, 2013) related to how their experiences of SVSH were handled, namely that their university failed to prevent sexual violence on campus through practices of inaction or omission, is complicit with instances of SVSH, and responds poorly when sexual violence occurs.

In our study, some graduate students did not know whether SVSH was a problem on their campus due to lack of data and transparency; others were unaware of what SVSH services their campus had to offer for graduate students specifically. Multiple studies explicate the challenges universities have with tailoring SVSH prevention and education efforts to their undergraduate populations (Karjane, Fisher, & Francis, 2002), publicizing existing services (Chiara & Lavina, 2014; Karjane et al., 2002; Potter et al., 2000), and having unclear or misleading information about policy and campus adjudication processes (Chiara & Lavina, 2014; Karjane et al., 2002; Krivoshey et al., 2013). The same evaluation of services for graduate students is un- or underdeveloped. This is a critical oversight that requires addressing to meet the needs of graduate students; those in our study reported experiences of isolation, lacking connection, and fewer social ties to the university and to their peers than undergraduate students. While addressing wellness and mental health among graduate students has become prioritized in academia and research (Grady et al., 2013; Ray et al., 2019; Scherr et al., 2020), we found that graduate students who lacked social connectedness and experienced isolation may have a false sense of security that they will not experience SVSH, at least not in the “stereotypical” ways that undergraduates do. This may lead graduate students to ignore or disengage with efforts the university *does* have in place to address SVSH.

It is notable that universities struggle to ensure that SVSH messaging is tailored to diverse student populations (Schulze & Perkins, 2017), and graduate students are noticeably absent in most of the literature surrounding perceptions and effectiveness of programming (Garcia et al., 2012; Vladutiu et al., 2011). The consideration of the varied places in which graduate students contend with SVSH (e.g., classrooms, laboratories, field settings) necessitates the design and/or revision of site-specific university policies to include graduate students, for both on-campus and off-campus contexts. Addressing the existing shortcomings and “expanding who is invited to the table” to combat sexual violence as McMahon et al. (2019) suggested through the use of a “Whole School Approach” framework (McMahon et al., 2019) is critical to better support all university students, including graduate students, given their unique vulnerabilities and fears, as we have addressed in this article. We hope our findings motivate university leaders and advocates to be more intentionally mindful of the ways that SVSH affects graduate students and to be more inclusive of who participates in policy-making, resource design, and care for survivors.

Graduate students reflected on the problematic power dynamics that exist between themselves and the faculty with whom they work—power dynamics that may arise from the liminal space in which graduates exist as students, workers, and emerging scholars. Limited research on this topic has been published since the 1980s (McKinney et al., 1988; Sandler & Hall, 1986; Schneider, 1987), when graduate students—specifically women, students of color, and queer students—began to come forward with issues akin to what our study highlights. As demonstrated in graduate students’ narratives, graduate students’ dependence on faculty for financial support, letters of recommendation during and after graduate school, and other forms of access to networking necessary for career growth may amplify the risk of abuse by faculty. As the limited peer-reviewed literature has demonstrated (Cantalupo & Kidder, 2017; Cholewinski & Burge, 1990; Singer, 1989) and as numerous cases highlighted in the media and grey literature in the last five years have shown, faculty found to have engaged in “consensual” relationships with their graduate students often face only a slap on the wrist, if there are repercussions at all. However, the students who report them often experience bullying, blackballing, lawsuits, and are even pushed out of their programs or from academia altogether (Flaherty, 2019; Greenberg, 2018; Kelsky, 2017; Schwartz & Abrams, 2020). A recent report on sexual harassment in academia (Johnson et al., 2018) demonstrates that when bullying, racism, xenophobia, and other inappropriate social behaviors are tolerated, the academy becomes a breeding ground for other forms of violence, including SVSH. It is unsurprising, then, that so many graduate students discussed their fears about reporting, while others reflected on the multitude of negative consequences they or their peers endured when they chose to use university reporting processes, which are purportedly meant to protect and support students. More concerted efforts are needed by our academic institutions to both increase accountability for faculty and address the fear of its students in reporting and accessing resources.

Given a recent study finding that college graduates do not believe their universities take discrimination complaints seriously (Marken, 2020), universities and academic communities more broadly have a long way to go to secure equity and safety on campus and gain the confidence of their students. While there is not a “one-size fits-all” solution, a handful of universities have been increasingly successful in creating institutional shifts within their unique university settings, including Georgia State University’s “Real Consent” program and Columbia University’s SHIFT study (Student Health Promotion, 2020; The Sexual Health Initiative to Foster Transformation, 2019). A component of building institutional trust includes hiring and retaining truly representative and diverse groups of faculty, staff and students (including male faculty) who are actively engaged in SVSH research and advocacy (Orenstein, 2020), comprehensive and exhaustive training of university staff and those responsible for SVSH prevention, mitigation (e.g., service providers, campus security), and thinking about diversity and inclusion as inseparable from violence prevention (Fotheringham et al., 2020; Marine & Nicolazzo, 2017; Terrazas-Carillo et al., 2019). Institutional culture change is rooted in shared accountability. Altogether, such efforts can help reimagine how SVSH prevention and support for survivors is offered on university campuses and reframe institutional care for survivors alongside compassionate visibility of survivorship. However, currently these steps forward are geared toward the undergraduate experience, leaving graduate students’ unique needs and interests frequently unattended to. This article is an attempt to address and redirect efforts to strengthen services and support provided for graduate students and, at the bare minimum, also include graduate students in these efforts.

## Conclusion

There have long been calls to reform and shift the culture of academia and to “unsilence the violence” (Gialopsos, 2017), including the implementation of the Clery Act and Title IX, which attempted to create mechanisms for SVSH-related transparency, accountability and prevention (Cruz, 2020; Perkins & Warner, 2017). With multiple federal, university, and student-led initiatives, programs and grassroots activism, there is underlying hope and messaging that SVSH *is* preventable on university campuses; however, a long history exists of silencing students, both intentionally and unintentionally, and counteracts significant progress being made toward preventing violence before it occurs. As Gialopsos (2017) posits, “prevention needs to be theoretically relevant, empirically driven and tailored” to specific campuses and student populations. This includes reexamining and recreating policies, prevention measures, campus climate surveys and resources so that they include the entire campus community (McMahon et al., 2019), are gender-neutral, no longer heteronormative (Gialopsos, 2017), and focus on the underlying systemic issues that continue to propagate violence instead of dismantling it. It also includes incorporating and prioritizing graduate student voices. In an April 2020 memo to the UC Provost, UC Academic Council Chair Dr. Kum-Kum Bhavnani wrote that “Graduate education and graduate students are the ‘soul’ of a university: the often invisible yet central aspect for all research universities. Support for them… is critical” (Bhavnani, 2020). It is imperative that universities include graduate students in SVSH prevention and education, that their unique vulnerabilities to SVSH are acknowledged and addressed, and that academic institutions address their shortcomings in holding faculty and staff accountable for perpetrating SVSH and maintaining a culture of silence about it in the academy; these recommendations are but a start.

## Limitations and Future Directions

Qualitative studies have a number of unique strengths, including the ability to examine understudied populations and issues with detail and depth; however, there are also limitations to qualitative study design. Due to the sensitive nature of the interviews and the general fear that graduate students experience about reporting unfavorable or harmful details about those they work with or around, there may have been instances of social desirability bias or self-censoring, especially in focus group discussions. To mitigate the risk of social desirability influencing our analysis, we utilized interviews *and* focus groups to capture both individual- and group-level perspectives. Future studies should consider using similar approaches to minimize such risk. Additionally, in this study we used non-randomized stratified sampling methods (including snowball sampling), which allowed us to secure increased participation from key student groups who are historically difficult to recruit (LGBTQ + students, students of color), especially in graduate school settings, where such student groups are admitted and retained at lower rates than undergraduates (Mack et al., 2005; Naderifar et al., 2017). While using these methods may have biased our sample towards those interested in SVSH or those who had experienced SVSH, we were able to hear from many diverse student voices. Nevertheless, our participants were generally white, cisgender and heterosexual and were likely not representative of all graduate students in the UC system or of graduate students in general. More concerted efforts must be made to capture the experiences of graduate students who may not outwardly appear at increased risk of SVSH, including international students, students with disabilities and Deferred Action for Childhood Arrivals (DACA) students.

In many ways, this is a first-look investigation of how graduate students are uniquely vulnerable to SVSH and how their experiences are shaped by the multiple roles they play in the university system. More research is necessary in a multitude of graduate student programs including smaller schools, private institutions, graduate-only institutions, and professional training programs (e.g., medical school, law school). We advocate for further efforts to accurately and adequately capture the prevalence of SVSH on university campuses and to further our understanding of the unique vulnerabilities and experiences of graduate students. SVSH within the academic institution *is* preventable; however, this will not and cannot happen until concerted efforts are made toward shifting the culture of academia, including the integration of diversity and intersectionality into violence-prevention work; inclusion of graduate students as SVSH researchers and policy decision-makers; and holding university leaders accountable for the policies, procedures and practices that protect perpetrators and harm survivors.
